# Association Between the Duration of Elevated Perfusion Pressure and Neurological Outcomes in Out-of-Hospital Cardiac Arrest Survivors

**DOI:** 10.31083/RCM42733

**Published:** 2025-12-19

**Authors:** Dong Hun Lee, Seok Jin Ryu, Byung Kook Lee, Yong Hun Jung, Kyung Woon Jeung, Hyo Jin Bang, Hyo Jeong Kwon, Joo Suk Oh, In Soo Cho

**Affiliations:** ^1^Department of Emergency Medicine, Chonnam National University Medical School, 61469 Gwangju, Republic of Korea; ^2^Department of Emergency Medicine, Chonnam National University Hospital, 61469 Gwangju, Republic of Korea; ^3^Department of Emergency Medicine, Seoul St. Mary's Hospital, College of Medicine, The Catholic University of Korea, 06591 Seoul, Republic of Korea; ^4^Department of Emergency Medicine, Asan Medical Center, University of Ulsan College of Medicine, 05505 Seoul, Republic of Korea; ^5^Department of Emergency Medicine, Uijeongbu St. Mary's Hospital, College of Medicine, The Catholic University of Korea, 06591 Seoul, Republic of Korea; ^6^Department of Emergency Medicine, KEPCO Medical Center, 01450 Seoul, Republic of Korea

**Keywords:** cardiac arrest, neurological outcomes, mean arterial pressure, targeted temperature management

## Abstract

**Background:**

The association between elevated perfusion pressure and neurological outcomes in out-of-hospital cardiac arrest (OHCA) survivors remains unclear. Specifically, to our knowledge, no studies have currently investigated whether the duration of elevated perfusion pressure influences neurological prognosis following OHCA. Thus, this study aimed to examine the association between the duration of a mean arterial pressure (MAP) >80 mmHg during the first 48 hours after return of spontaneous circulation (ROSC) and neurological outcomes in OHCA survivors.

**Methods:**

This observational study included adult patients (≥18 years) with OHCA treated between January 2019 and May 2021. The cumulative duration of a MAP >80 mmHg was recorded during the 0–24, 25–48, and 0–48 hour intervals following ROSC. The primary outcome was the neurological status at 6 months, with good outcomes defined as Cerebral Performance Category (CPC) scores of 1 or 2.

**Results:**

Among the 468 patients with OHCA, 132 (28.2%) achieved good neurological outcomes. The duration of a MAP >80 mmHg over 0–48 hours was significantly longer in the good outcome group compared with the poor outcome group (35 (26–42) vs. 28 (16–39) hours; *p* < 0.001). In the multivariable analysis after adjusting for confounders, longer durations of a MAP >80 mmHg at 0–48 hours (odds ratio (OR): 1.047, 95% confidence interval (CI): 1.021–1.073) and 25–48 hours (OR: 1.086, 95% CI: 1.042–1.131), but not at 0–24 hours, were associated with good neurological outcomes at 6 months.

**Conclusions:**

The duration of a MAP >80 mmHg during the 0–48 and 25–48 hour periods after ROSC was associated with good neurological outcomes at six months in OHCA survivors.

## 1. Introduction

Even after the return of spontaneous circulation (ROSC) in patients with out-of-hospital cardiac arrest (OHCA), comprehensive post-cardiac arrest care is critical to mitigating ongoing ischemia–reperfusion injury [1, 2, 3]. Maintaining arterial pressure (AP) above a certain threshold is essential to ensure adequate organ and tissue perfusion. In the post-ROSC phase, mean AP (MAP) plays a key role in supporting cerebral perfusion and has been associated with overall patient prognosis [4, 5]. Adequate MAP is crucial for preserving oxygen delivery to the brain and other vital organs, minimizing secondary ischemic damage, and facilitating neurological recovery.

To ensure sufficient tissue perfusion, preserve renal function (urine output), and stabilize metabolic processes (e.g., lactate clearance), current international guidelines recommend maintaining MAP at ≥65 mmHg [4, 5]. However, several studies have suggested that targeting a MAP above this threshold after ROSC may be associated with improved neurological outcomes [6, 7, 8, 9]. By contrast, a randomized clinical trial found no significant difference in clinical outcomes between patients managed with a MAP of 65–75 mmHg and those managed with 80–100 mmHg, using 80 mmHg as the comparative threshold [10]. Notably, that study excluded patients with unwitnessed arrests, non-shockable rhythms, and non-cardiac etiologies [10], limiting its generalizability to the broader OHCA population. As a result, it is likely that the study cohort primarily comprised patients with milder disease severity, potentially contributing to the reported favorable prognosis (>60%) [10]. Additionally, while previous studies have assessed average MAP during the observation period [6, 7, 8, 9], none have investigated the relationship between the duration of exposure to elevated MAP and neurological outcomes following ROSC.

The present study quantified the duration of MAP >80 mmHg within the first 48 hours after ROSC and assessed its association with neurological outcome groups in OHCA survivors. Furthermore, using the revised post-Cardiac Arrest Syndrome for Therapeutic hypothermia score (rCAST) as a measure of illness severity [11, 12], we evaluated whether this association varied according to the severity of injury following cardiac arrest.

## 2. Materials and Methods

### 2.1 Study Design and Population

The Korean Hypothermia Network (KORHN) established a prospective, multicenter registry of comatose adult (≥18 years) survivors of OHCA who underwent targeted temperature management (TTM) at 28 participating hospitals beginning in October 2015 (KORHN-Prospective Registry [KORHN-PRO 1.0], NCT02827422). Between January 2019 and May 2021, at 12 of these institutions, the registry was expanded to include hourly blood pressure measurements and arterial blood gas analyses every four hours within the first 48 hours after ROSC. The study protocol was approved by the institutional review boards of all participating centers. Written informed consent was obtained from all patients or their legally authorized representatives, in accordance with national regulations and the principles outlined in the Declaration of Helsinki [13].

A retrospective analysis was conducted using data from the KORHN-PRO registry. Eligible participants were comatose adult OHCA patients who received TTM. Patients were excluded if they lacked data on Sequential Organ Failure Assessment (SOFA) scores, rCAST scores, blood pressure recordings for more than six hours within the first 48 hours post-ROSC, or 6-month neurological outcomes.

### 2.2 Targeted Temperature Management and Blood Pressure Management

All enrolled comatose OHCA survivors received TTM. A feedback-controlled cooling system was employed to maintain a target temperature of 33–36 °C for 24 hours. To prevent shivering and provide sedation, patients received propofol, midazolam, or remifentanil for analgosedation. Following the maintenance phase, rewarming was initiated at a controlled rate of 0.25 °C per hour. All other aspects of post-arrest care were managed according to institutional protocols, consistent with international guidelines [14]. Systolic and diastolic blood pressures were measured via invasive arterial catheters, with continuous arterial pressure monitoring. MAP was calculated, and vasopressors and fluids were administered to maintain MAP ≥65 mmHg in accordance with clinical guidelines [14].

### 2.3 Data Collection

The following data were extracted from the registry: age, sex, body mass index, preexisting comorbidities, witnessed arrest status, presence of bystander cardiopulmonary resuscitation, initial cardiac rhythm, cardiac arrest etiology, time from collapse to ROSC, SOFA score within 24 hours post-ROSC [15], arterial pH, Glasgow Coma Scale motor response score, serum lactate concentration after ROSC, systolic and diastolic blood pressure (measured hourly post-ROSC), and 6-month outcomes based on the Cerebral Performance Category (CPC) scale.

The duration for which MAP exceeded 80 mmHg during the first 48 hours after ROSC was calculated and stratified into three intervals: 0–24 hours, 25–48 hours, and 0–48 hours. The rCAST score was computed based on previously described clinical variables [11, 12], and patients were classified into low (≤5.5), moderate (6.0–14.0), or high (≥14.5) severity groups accordingly.

Neurological outcomes were assessed at 6 months post-arrest using the CPC scale, via structured telephone interviews with the patient, or when this was not feasible, with a caregiver or legal proxy. CPC outcomes were categorized as follows: CPC 1 (good performance), CPC 2 (moderate disability), CPC 3 (severe disability), CPC 4 (vegetative state), and CPC 5 (brain death or death) [16].

### 2.4 Statistical Analyses

Categorical variables are presented as frequencies with corresponding percentages, and comparisons between neurological outcome groups were performed using the chi-square test or Fisher’s exact test, as appropriate. Continuous variables are reported as medians with interquartile ranges (IQRs). Given that all continuous variables were non-normally distributed, the Mann–Whitney U test was applied for comparisons between groups.

To explore the association between the duration of MAP >80 mmHg and neurological outcomes, logistic regression analysis was performed. Variables with *p* < 0.2 in univariate analysis were initially considered for inclusion in the multivariable model. However, variables such as witnessed arrest, shockable rhythm, lactate level, and time from collapse to ROSC were excluded from the multivariable model because these factors are already integrated within the rCAST score. A backward stepwise selection method was employed, with sequential elimination of variables using a *p*-value threshold of >0.10 to construct the final adjusted model. The final covariates included age, male sex, cardiac etiology, SOFA score, and rCAST score (**Supplementary Table 1**). To assess the independent association, each MAP duration variable (>80 mmHg during 0–24, 24–48, and 0–48 hours) was analyzed separately in the final model. Results are presented as adjusted odds ratios (ORs) with 95% confidence intervals (CIs). Statistical significance was defined as a two-sided *p* < 0.05. All analyses were conducted using SPSS Statistics version 26.0 for Windows (IBM Corp., Armonk, NY, USA).

## 3. Results

### 3.1 Patient Characteristics

**Fig. 1.**
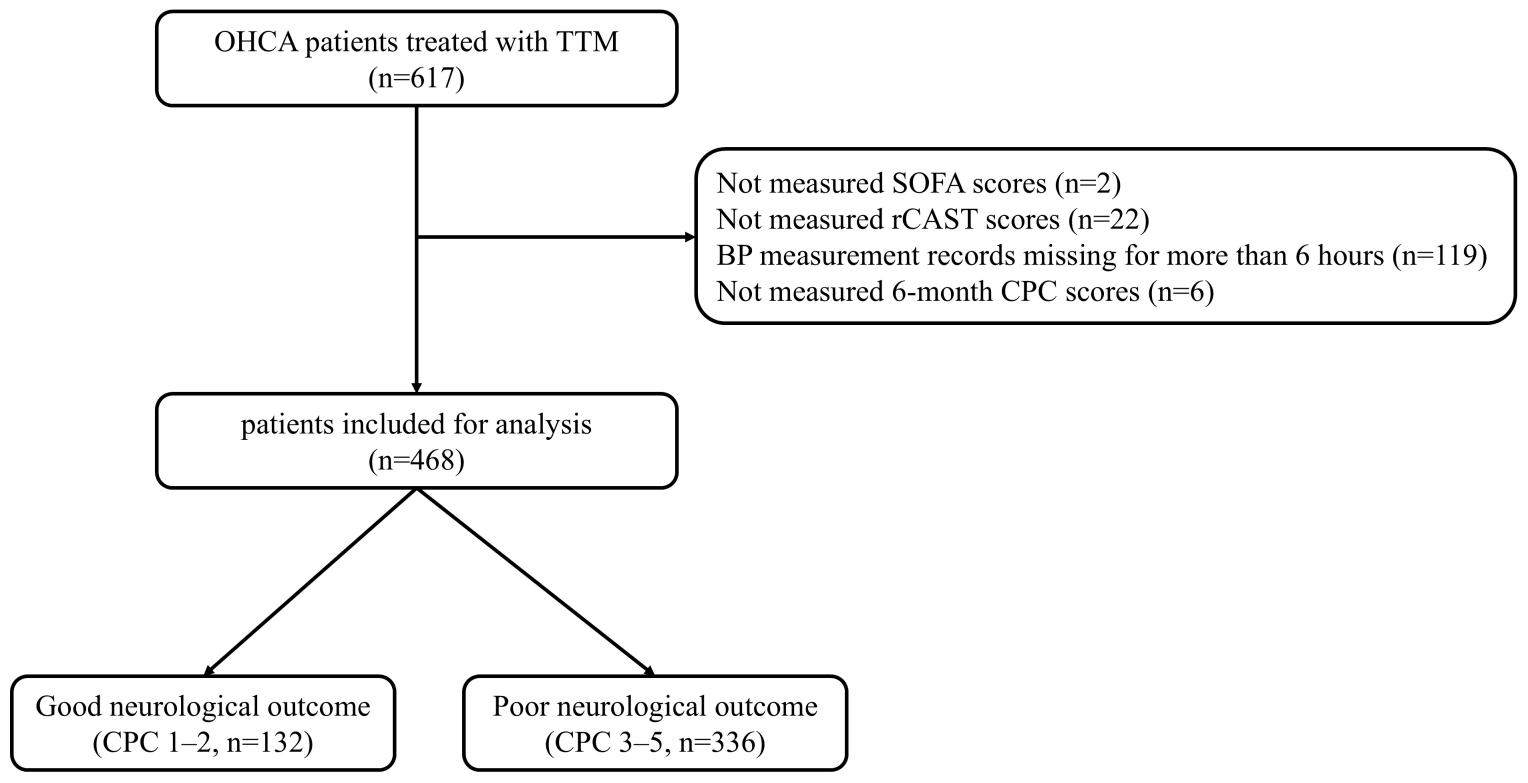
**Flow diagram of patient inclusion**. OHCA, out-of-hospital cardiac arrest; TTM, targeted temperature management; SOFA, Sequential Organ Failure Assessment; rCAST, revised post-Cardiac Arrest Syndrome for Therapeutic hypothermia score; CPC, Cerebral Performance Category.

Of the 617 OHCA survivors recorded in the registry, patients were excluded if SOFA scores could not be assessed (n = 2), rCAST scores could not be calculated (n = 22), blood pressure recordings were unavailable for more than 6 hours within the first 48 hours after ROSC (n = 119), or 6-month CPC scores were missing (n = 6). Ultimately, 468 patients were included in the final analysis (Fig. 1). **Supplementary Table 2** shows the results of comparison of characteristics between the excluded and included patients. There were significant differences in proportion of malignancy between the included and excluded patients (4.7% vs. 10.1%; *p* = 0.027). There was no significant difference in the time from neurologic outcome between the two groups.

Patients were stratified into good (n = 132, 28.2%) and poor (n = 336, 71.8%) neurological outcome groups based on CPC scores at 6 months (Table 1). Compared with those in the poor outcome group, patients in the good outcome group were younger, more frequently male, and had higher body mass indexes. They also had a greater prevalence of coronary artery disease and a lower prevalence of diabetes. Regarding cardiac arrest characteristics, the good outcome group exhibited higher rates of witnessed arrests and shockable rhythms, as well as shorter times from collapse to ROSC. Additionally, this group had lower post-ROSC lactate levels, SOFA scores, and rCAST scores. The duration of MAP >80 mmHg was longer in the good outcome group across all time intervals: 0–24 hours (17 vs. 15 hours), 0–48 hours (35 vs. 28 hours), and 25–48 hours (18 vs. 12 hours). There was no significant difference in targeted temperature distribution according to neurological outcome at 6 months.

**Table 1.** **Comparisons of baseline characteristics based on neurological outcomes at 6 months**.

Variables		Total (n = 468)	Good (n = 132)	Poor (n = 336)	*p*
Demographics					
	Age (years), median (IQR)	61.3 (49.3–71.8)	56.2 (47.6–65.5)	64.0 (50.1–75.2)	<0.001
	Male, n (%)	331 (70.7)	111 (84.1)	220 (65.5)	<0.001
	Body mass index (kg/m^2^), median (IQR)	23.5 (21.3–25.6)	24.2 (22.4–26.3)	23.1 (20.5–25.2)	<0.001
Preexisting illness, n (%)					
	Coronary artery disease	60 (12.8)	26 (19.7)	34 (10.1)	0.005
	Arrhythmia	24 (5.1)	9 (6.8)	15 (4.5)	0.420
	Congestive heart failure	21 (4.5)	5 (3.8)	16 (4.8)	0.834
	Hypertension	202 (43.2)	51 (38.6)	151 (44.9)	0.256
	Diabetes	142 (30.3)	25 (18.9)	117 (34.8)	<0.001
	Stroke	41 (8.8)	8 (6.1)	33 (9.8)	0.266
	Previous pulmonary disease	38 (8.0)	5 (3.8)	33 (9.8)	0.050
	Previous renal disease	42 (9.0)	7 (5.3)	35 (10.4)	0.118
	Liver cirrhosis	8 (1.7)	1 (0.8)	7 (2.1)	0.451
	Malignancy	22 (4.7)	6 (4.5)	16 (4.8)	0.999
Cardiac arrest characteristics					
	Witnessed collapse, n (%)	302 (64.5)	96 (72.7)	206 (61.3)	0.027
	Bystander CPR, n (%)	321 (68.6)	97 (73.5)	224 (66.7)	0.187
	Shockable rhythm, n (%)	161 (34.4)	98 (74.2)	63 (18.8)	<0.001
	Cardiac etiology, n (%)	257 (54.9)	106 (80.3)	151 (44.9)	<0.001
	Time from collapse to ROSC (min), median (IQR)	29.0 (17.0–46.0)	17.0 (12.0–27.0)	35.0 (20.0–50.0)	<0.001
Lactate after ROSC (mmol/L), median (IQR)		9.2 (5.9–12.2)	6.7 (4.0–9.5)	10.2 (6.9–12.9)	<0.001
SOFA score		11 (9–13)	10 (8–12)	12 (10–13)	<0.001
rCAST		13 (8–16)	7 (3–10)	15 (12–16)	<0.001
Duration of MAP >80 mmHg					
	During 0–48 h (hour), median (IQR)	30 (18–40)	35 (26–42)	28 (16–39)	<0.001
	During 0–24 h (hour), median (IQR)	16 (11–20)	17 (13–21)	15 (10–20)	0.009
	During 25–48 h (hour), median (IQR)	14 (6–21)	18 (13–22)	12 (5–20)	<0.001

IQR, interquartile range; CPR, cardiopulmonary resuscitation; ROSC, return of spontaneous circulation; SOFA, Sequential Organ Failure Assessment; rCAST, revised post-Cardiac Arrest Syndrome for Therapeutic hypothermia score; MAP, mean arterial pressure.

**Fig. 2.**
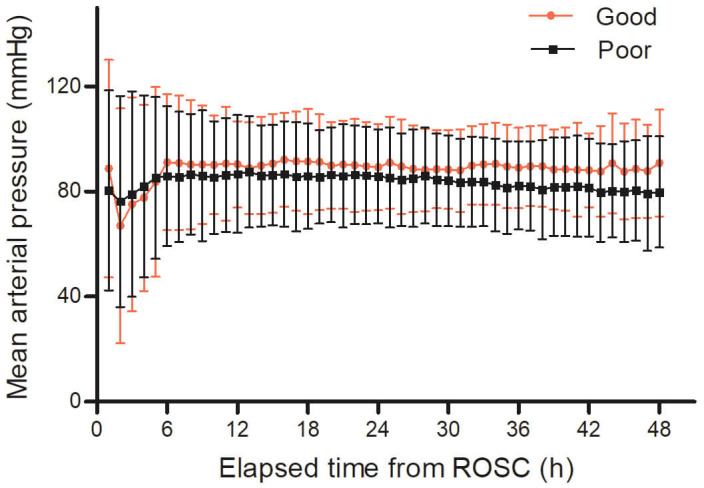
**Hourly MAP within 48 hours after ROSC based on neurological outcomes**. MAP, mean arterial pressure; ROSC, return of spontaneous circulation.

Fig. 2 shows hourly MAP values during the first 48 hours after ROSC. Patients with good neurological outcomes exhibited consistently higher MAPs throughout the 48-hour period compared with those with poor outcomes. The difference in MAP between the two groups became more pronounced during the 25–48 hour interval after ROSC (Fig. 2).

### 3.2 Comparison of Baseline Characteristics According to rCAST Score

Based on rCAST severity, patients were categorized into low (n = 71, 15.2%), moderate (n = 215, 45.9%), and high (n = 182, 38.9%) severity groups (Table 2). Statistically significant differences were observed across the three groups with respect to sex distribution, prevalence of coronary artery disease, witnessed collapse, bystander cardiopulmonary resuscitation, shockable rhythm, cardiac etiology, time from collapse to ROSC, lactate levels, SOFA scores, and rCAST scores. However, no significant differences were found in the duration of MAP >80 mmHg during the 0–24, 0–48, or 25–48 hour intervals (Table 2).

**Table 2.** **Comparison of baseline characteristics according to rCAST severity**.

Variables		Low severity (n = 71)	Moderate severity (n = 215)	High severity (n = 182)	*p*
Demographics					
	Age (years), median (IQR)	58.5 (48.8–65.8)	63.1 (51.3–74.3)	61.9 (46.9–72.0)	0.148
	Male, n (%)	58 (81.7)	153 (71.2)	120 (65.9)	0.046
	Body mass index (kg/m^2^), median (IQR)	23.7 (21.6–25.6)	23.4 (21.3–25.7)	23.7 (20.6–25.5)	0.935
Preexisting illness, n (%)					
	Coronary artery disease	14 (19.7)	33 (15.3)	13 (7.1)	0.007
	Arrhythmia	4 (5.6)	10 (4.7)	10 (5.5)	0.882
	Congestive heart failure	5 (7.0)	12 (5.6)	4 (2.2)	0.127
	Hypertension	23 (32.4)	103 (47.9)	76 (41.8)	0.064
	Diabetes	14 (19.7)	69 (32.1)	59 (32.4)	0.102
	Stroke	5 (7.0)	23 (10.7)	13 (7.1)	0.462
	Previous pulmonary disease	4 (5.6)	18 (8.4)	16 (8.8)	0.762
	Previous renal disease	1 (1.8)	25 (11.6)	16 (8.8)	0.019
	Liver cirrhosis	2 (2.8)	2 (0.9)	4 (2.2)	0.337
	Malignancy	4 (5.6)	12 (5.6)	6 (3.3)	0.484
Cardiac arrest characteristics					
	Witnessed collapse, n (%)	65 (91.5)	154 (71.6)	83 (45.6)	<0.001
	Bystander CPR, n (%)	51 (71.8)	167 (77.7)	103 (58.6)	<0.001
	Shockable rhythm, n (%)	57 (80.3)	80 (37.2)	24 (13.2)	<0.001
	Cardiac etiology, n (%)	58 (81.7)	132 (61.4)	67 (36.8)	<0.001
	Time from collapse to ROSC (min), median (IQR)	16.0 (12.0–20.0)	24.0 (15.0–39.0)	42.0 (30.0–54.3)	<0.001
Lactate after ROSC (mmol/L), median (IQR)		4.6 (3.5–7.9)	8.0 (5.6–10.4)	12.1 (9.8–15.0)	<0.001
SOFA score		10 (7–11)	11 (9–13)	12 (10–13)	<0.001
rCAST		3 (2–5)	11 (9–13)	16 (16–18)	<0.001
Duration of MAP >80 mmHg					
	During 0–48 h (hour), median (IQR)	32 (22–40)	30 (19–39)	30 (17–41)	0.315
	During 0–24 h (hour), median (IQR)	16 (11–20)	16 (10–20)	16 (11–20)	0.828
	During 25–48 h (hour), median (IQR)	17 (10–22)	14 (6–21)	13 (5–22)	0.130
Poor neurologic outcome, n (%)		14 (19.7)	148 (68.8)	174 (95.6)	<0.001

IQR, interquartile range; CPR, cardiopulmonary resuscitation; ROSC, return of spontaneous circulation; SOFA, Sequential Organ Failure Assessment; rCAST, revised post-Cardiac Arrest Syndrome for Therapeutic hypothermia score; MAP, mean arterial pressure.

In subgroup analyses based on outcome, patients in the low severity group with good outcomes had a longer MAP >80 mmHg duration during the 25–48 hour interval (18 vs. 12 hours), while durations at 0–24 and 0–48 hours did not differ significantly between outcome groups (**Supplementary Table 3**). Among patients in the moderate severity group, those with good outcomes had longer MAP >80 mmHg durations at 0–24 (17 vs. 15 hours), 25–48 (18 vs. 12 hours), and 0–48 (34 vs. 27 hours) hours compared to those with poor outcomes. In the high severity group, patients with good outcomes had a longer MAP >80 mmHg duration at 0–48 hours (41 vs. 30 hours), whereas no significant differences were observed for the 0–24 or 25–48 hour intervals (**Supplementary Table 3**).

### 3.3 Multivariable Analysis of the Duration of MAP >80 mmHg for Good Neurologic Outcome at 6 Months

After adjusting for confounders, multivariable logistic regression analysis revealed that the duration of MAP >80 mmHg was significantly associated with good neurological outcomes at 6 months when measured during the 25–48 hour (OR: 1.086, 95% CI: 1.042–1.131) and 0–48 hour (OR: 1.047, 95% CI: 1.021–1.073) intervals. No significant association was observed for the 0–24 hour interval (OR: 1.041, 95% CI: 0.989–1.097) (Table 3).

**Table 3.** **Multivariable analysis of the duration of MAP at >80 mmHg for good neurological outcomes at 6 months**.

Variables		Adjusted OR (95% CI) ^a^	*p*
Duration of MAP >80 mmHg			
	During 0–48 h, hour	1.047 (1.021–1.073)	<0.001
	During 0–24 h, hour	1.041 (0.989–1.097)	0.125
	During 25–48 h, hour	1.086 (1.042–1.131)	<0.001

Each variable was individually entered into the final model and analyzed separately. ^a^Adjusted for age, male sex, cardiac etiology, SOFA score, and rCAST score. MAP, mean arterial pressure; OR, odds ratio; CI, confidence interval; SOFA, Sequential Organ Failure Assessment; rCAST, revised post-Cardiac Arrest Syndrome for Therapeutic hypothermia score.

Table 4 presents stratified multivariable analyses of MAP >80 mmHg duration by rCAST severity group. In the moderate severity group, the duration of MAP >80 mmHg remained significantly associated with good neurological outcomes during both the 25–48 hour (OR: 1.094, 95% CI: 1.036–1.154; *p* < 0.001) and 0–48 hour (OR: 1.044, 95% CI: 1.011–1.079; *p* = 0.008) intervals. No significant association was observed for the 0–24 hour interval. By contrast, in both the low and high severity groups, no statistically significant association was observed between the duration of MAP >80 mmHg and neurological outcomes across any of the time intervals examined (Table 4).

**Table 4.** **Multivariable analysis of the duration of MAP >80 mmHg for good neurologic outcome according to rCAST severity**.

Variables		Low severity (n = 71)		Moderate severity (n = 215)		High severity (n = 182)	
		Adjusted OR (95% CI) ^a^	*p*	Adjusted OR (95% CI) ^b^	*p*	Adjusted OR (95% CI) ^c^	*p*
Duration of MAP >80 mmHg							
	During 0–48 h, hour	1.043 (0.987–1.103)	0.137	1.044 (1.011–1.079)	0.008	1.074 (0.999–1.154)	0.055
	During 0–24 h, hour	1.074 (0.959–1.202)	0.218	1.038 (0.976–1.103)	0.237	1.110 (0.953–1.294)	0.179
	During 25–48 h, hour	1.075 (0.978–1.182)	0.132	1.094 (1.036–1.154)	<0.001	1.103 (0.991–1.227)	0.072

^a^ Adjusted by body mass index, presence of stroke, and presence of previous pulmonary disease. ^b^ Adjusted by age, male, diabetes, cardiac etiology, and rCAST score. ^c^ Adjusted by age and presence of coronary artery disease. MAP, mean arterial pressure; rCAST, revised post-cardiac arrest syndrome for therapeutic hypothermia score; OR, odds ratio; CI, confidence interval.

## 4. Discussion

This study demonstrated that the duration of MAP >80 mmHg during the 25–48 and 0–48 hour intervals, but not during the initial 0–24 hours, was associated with good neurological outcomes at 6 months following ROSC. Notably, among patients classified in the moderate severity category based on rCAST scores, the association between MAP >80 mmHg duration and good neurological outcomes was observed during both the 25–48 and 0–48 hour periods.

Previous research has reported that cerebral autoregulation is often impaired in patients with post-cardiac arrest, with a rightward shift in the lower limit of autoregulation. Specifically, patients resuscitated from cardiac arrest exhibited a significantly higher threshold for maintaining cerebral perfusion (114 mmHg) compared to healthy controls (76 mmHg) [17]. These findings suggest that a higher-than-normal MAP may be required to ensure adequate cerebral perfusion in this population. Additional studies using brain tissue regional oxygen saturation identified mean optimal MAP thresholds exceeding 76 mmHg [18] and 89 mmHg [19]. Kilgannon *et al*. [7] further reported a threshold effect, wherein MAP values above 70 mmHg were associated with favorable neurological outcomes. Similarly, an observational study identified the MAP range of 76–86 mmHg as optimal for maximizing survival in cardiac arrest survivors [8], while another study linked a MAP >90 mmHg during the first 6 hours after ROSC to both improved survival and better neurological outcomes [9].

In our study, the threshold of MAP >80 mmHg was selected based on prior randomized controlled trials, including those by Jakkula *et al*. [10] (MAP 65–75 mmHg vs. 80–100 mmHg) and Kjaergaard *et al*. [20] (MAP 63 mmHg vs. 77 mmHg), which commonly used approximately 80 mmHg as the higher target range for post-cardiac arrest care. Although this threshold does not align with the clinical definition of hypertension, it was applied in this study to represent relatively elevated perfusion pressures. The observed association between sustained MAP >80 mmHg and favorable neurological outcomes may be attributable to its role in preserving cerebral microvascular perfusion. The no-reflow phenomenon—wherein microvascular obstruction persists despite restoration of large-vessel flow—has been described in both cardiac and cerebral ischemia. Kloner *et al*. [21] proposed that microvascular impairment resulting from endothelial swelling, pericyte constriction, and interstitial edema may hinder capillary perfusion after ischemia–reperfusion injury. Maintaining optimal MAP levels may therefore help sustain perfusion across damaged microvascular beds, potentially reducing secondary brain injury and promoting neurological recovery. Our findings support this mechanism, demonstrating that longer durations of MAP >80 mmHg were associated with good neurological outcomes.

Our findings demonstrate an association between the duration of MAP >80 mmHg and favorable neurological outcomes specifically during the 25–48 hour interval, but not during the initial 0–24 hours. As illustrated in Fig. 2, both neurological outcome groups exhibited hemodynamic stabilization following the initial resuscitation phase; however, patients in the good outcome group consistently maintained higher MAP values throughout the 48-hour observation period. Notably, during the 25–48 hour interval, the MAP declined in the poor outcome group, leading to a widening divergence between the groups. This growing disparity in MAP may partly account for the differential associations observed between MAP duration and neurological outcomes. Previous studies have identified that hemodynamic instability is typically most pronounced around 6 hours post-ROSC, corresponding to the lowest observed cardiac index and MAP during the early resuscitation phase [22, 23]. While the precise timing of nadir MAP varies slightly across studies, both consistently reported minimal cardiac output at approximately 6 hours, followed by gradual improvement in MAP beyond 24 hours [22, 23]. Concurrently, this period is also characterized by peak hypoperfusion and secondary brain injury [24, 25]. Although moderate hypothermia during TTM may suppress cerebral metabolic demands and attenuate the inflammatory cascade, the rewarming phase can paradoxically increase metabolic requirements [26] and exacerbate inflammation [27], thereby aggravating secondary neurological injury. Thus, the stronger association between MAP and neurological outcomes during the 25–48 hour period may be attributable to evolving hemodynamic stress linked to these inflammatory processes. This timeframe represents a critical convergence of multiple concurrent pathophysiological mechanisms: the persistently elevated autoregulation threshold (requiring MAP substantially above traditional targets for adequate perfusion) [17], the transition from hypothermic cerebral metabolic suppression to progressively increasing metabolic demands [26], and potential inflammatory cascade activation during rewarming [27]. The temporal coincidence of impaired cerebral autoregulation with rising metabolic requirements and inflammatory burden during the 24–48 hour interval provides a pathophysiological basis for the observed association between MAP >80 mmHg maintenance and improved neurological outcomes during this specific period. Collectively, these findings underscore the potential importance of sustaining adequate perfusion and systemic recovery beyond the initial resuscitative window.

Our multivariable analysis revealed strong independent associations between male sex (OR: 2.427, 95% CI: 1.220–4.827) and cardiac etiology (OR: 2.495, 95% CI: 1.290–4.825) with good neurological outcomes. The association between male sex and good neurological outcomes reflects multifactorial biological and clinical mechanisms. Recent meta-analyses encompassing over 1.2 million patients demonstrate that males present with shockable rhythms more frequently (39.6% vs. 25.7% in females), with meta-regression analysis showing initial shockable rhythm as a significant predictor of survival outcomes (*p* < 0.001) [28]. This rhythm disparity confers critical prognostic importance, as an initial shockable rhythm is regarded as one of the most important factors associated with survival after OHCA [29]. Furthermore, Bosson *et al*. [30] demonstrated that beyond favorable arrest characteristics, males receive more aggressive post-resuscitation interventions including coronary angiography (25% vs. 11%), percutaneous coronary intervention (PCI) (14% vs. 5%), and TTM (40% vs. 33%). Importantly, when these differences in treatment were accounted for in our analysis, the survival advantage for males disappeared, with no significant difference in neurological outcomes between males and females (OR: 0.9, 95% CI: 0.8–1.1) [30].

The association between cardiac etiology and good neurological outcomes in our study is consistent with established pathophysiological mechanisms. Unlike non-cardiac causes that involve prolonged hypoxia before arrest, cardiac arrests result from acute coronary occlusion with immediate circulatory cessation, limiting the extent of anoxic brain injury. The therapeutic reversibility of cardiac etiology is crucial—Dumas *et al*. [31] demonstrated that successful PCI in patients with significant coronary lesions improved survival from 31% to 51%. This finding is supported by nationwide data from Japan showing five-fold better neurological outcomes in cardiac versus non-cardiac origin OHCA (5.0% vs. 1.2%) among 547,153 patients [32]. These mechanisms—electrically reversible rhythms, shorter ischemic time, and treatable coronary pathology—collectively explain why cardiac etiology emerges as a powerful predictor of favorable neurological outcome, supporting aggressive interventional approaches in these patients.

Determining the optimal MAP target for post-cardiac arrest care remains challenging due to the complex interplay of ischemic brain injury, cerebral metabolic demands, and impaired autoregulation. This complexity may explain why recent randomized controlled trials have not demonstrated significant differences in neurological outcomes between patients managed with higher versus lower MAP thresholds [10, 20, 33]. Notably, these trials enrolled only patients with OHCA of presumed cardiac origin and excluded individuals with non-cardiac etiologies, who have worse prognoses [10, 20, 33]. By contrast, our study encompassed a broader patient population, with lower proportions of cardiac etiologies (54.9% vs. 100%) and shockable rhythms (34.4% vs. 100%, 66.7%, and 84.8%) compared with previous trials [10, 20, 33]. This wider inclusion likely contributed to the higher incidence of poor neurological outcomes observed in our cohort (71.8% vs. 35.0%, 63.2%, and 33.0%) [10, 20, 33]. These strict inclusion criteria in the randomized controlled trials (RCTs)—particularly the exclusion of non-cardiac etiologies and unwitnessed arrests—likely selected patients with relatively preserved cerebral autoregulation. Our broader inclusion criteria captured patients with more heterogeneous pathophysiology, where the therapeutic window for MAP optimization may be more relevant. This explains why the association between MAP duration and outcomes was most apparent in our moderate-severity subgroup, while previous RCTs with more homogeneous populations showed neutral results. Given the heterogeneity of our study population, including variations in cardiac arrest etiology and severity, we utilized the rCAST score to stratify injury severity and better evaluate the impact of MAP on neurological outcomes in OHCA survivors. The rCAST score has been extensively validated as a reliable prognostic tool in diverse OHCA populations. In a single-center U.S. validation study (n = 505), Kim *et al*. [34] demonstrated that the rCAST score achieved excellent discrimination for predicting poor neurological outcome (area under the curve [AUC]: 0.815; 95% CI: 0.763–0.867) and mortality (AUC: 0.799; 95% CI: 0.751–0.847), significantly outperforming the Pittsburgh Cardiac Arrest Category score for mortality prediction (*p* = 0.017). Similarly, in the multicenter study involving 658 patients across 24 intensive care units (ICUs), Lascarrou *et al*. [35] reported that rCAST maintained good performance (AUC: 0.82; 95% CI: 0.78–0.85), though it did not significantly outperform Utstein criteria (*p* = 0.16). Nevertheless, the authors emphasized the clinical utility of rCAST, noting its ease of calculation and rapid bedside determination as key advantages for routine implementation [35].

Our analysis showed that the association between the duration of MAP >80 mmHg and neurological outcomes was most evident during the 25–48 hour period in patients within the moderate severity group, compared with those in the low and high severity groups. These findings suggest that the effect of MAP on neurological outcomes may be modulated by the severity of illness in OHCA survivors. In our cohort, patients classified as having either low or high severity typically exhibited uniform neurological outcomes—either predominantly favorable or unfavorable. In contrast, the moderate severity group included a mixture of outcomes, indicating the presence of a “gray zone” in which the duration of MAP >80 mmHg may exert a meaningful influence on neurological prognosis. In the high-severity group, univariate analysis revealed a significant difference in the duration of MAP >80 mmHg between the good and poor prognosis groups (41 vs. 30 hours; *p* = 0.032), but not in multivariate analysis (*p* = 0.055). This discrepancy likely reflects the limited statistical power due to only eight patients achieving good outcomes in this subgroup, as well as the combined effects of severe neurological impairment that outweighed the benefits of maintaining MAP >80 mmHg. This difference in response by severity is consistent with the results of other post-cardiac arrest interventions. Prior observational studies have suggested that post-cardiac arrest injury severity, as measured by scoring systems or electroencephalography, is an important determinant of TTM effectiveness [11, 36, 37]. Notably, mild therapeutic hypothermia (33–34 °C) has been associated with improved neurological outcomes in moderately injured cardiac arrest survivors, whereas its benefits appear diminished in those with either minimal or severe injury [11, 37].

In patients with low severity, relatively intact cerebral autoregulation may render additional MAP elevation unnecessary. However, individuals with moderate severity are more likely to have impaired autoregulation and borderline cerebral perfusion, placing them within a physiological range where elevated MAP may meaningfully enhance oxygen delivery and mitigate secondary brain injury. Conversely, patients with high severity often have extensive, irreversible brain damage, limiting the potential benefit of elevated MAP. These observations underscore the value of tailoring post-resuscitation hemodynamic management based on individual injury severity. Identifying patients in the moderate injury category who may benefit from higher MAP targets could facilitate more personalized and effective post-cardiac arrest care.

This study had several limitations. First, as a retrospective observational study, it cannot establish causality between MAP duration and neurological outcomes. Second, although the use of a multicenter registry enhances generalizability, approximately 25% of patients were excluded due to missing data, potentially introducing selection bias. Third, MAP was recorded hourly rather than continuously. This intermittent sampling may have failed to capture transient hypotensive episodes between measurements, potentially contributing to secondary brain injury. While continuous monitoring would provide a more granular assessment of hemodynamic stability, our use of hourly data from a large, multicenter registry represents a pragmatic and methodological advance over many prior studies that relied on less frequent, averaged values. Fourth, our analysis did not account for other hemodynamic parameters that may influence cerebral perfusion and outcomes, a notable limitation of this study. Specific data on the dose and duration of vasopressors or inotropes, as well as on net fluid balance, were not included in our analysis. These interventions are significant potential confounders. For instance, higher vasopressor doses may indicate more severe post-cardiac arrest shock, an independent predictor of poor outcomes, while fluid management can affect both MAP and cerebral edema. Although our multivariable models adjusted for overall illness severity using SOFA and rCAST scores, these scores may not fully capture the influence of these specific pharmacologic interventions was not assessed in the current analysis, and the potential for residual confounding remains. Fifth, the primary outcome was assessed through telephone interviews with patients or proxies. This method may be less sensitive than direct examination for detecting subtle cognitive deficits. Proxy reporting is also susceptible to recall bias, potentially overestimating favorable outcomes. While this approach is pragmatic for multicenter studies, such misclassification could influence the observed associations. Finally, cerebral perfusion pressure was not measured, precluding direct assessment of its relationship with MAP.

## 5. Conclusions

In this study, duration of MAP >80 mmHg during the first 48 hours following ROSC was associated with good neurological outcomes. Further subgroup analysis indicated that this association was significant only during the 25–48 hour interval and was observed exclusively in patients with moderate injury severity, as defined by the rCAST score. No significant associations were found in the low or high severity groups.

## Availability of Data and Materials

All data generated or analyzed during this study are included in this article and its supplementary material files. Further enquiries can be directed to the corresponding author.

## Supplementary Material

Supplementary material associated with this article can be found, in the online version, at https://doi.org/10.31083/RCM42733.
