# Supplementary material for: Association Between the Duration of Elevated Perfusion Pressure and Neurological Outcomes in Out-of-Hospital Cardiac Arrest Survivors
Source: Rev Cardiovasc Med. 2025 Dec 19;26(12):42733. doi: 10.31083/RCM42733 (PMC12780993; doi:10.31083/RCM42733)
Supplement: Supplementary file 1 [file 2153-8174-26-12-42733-s1.docx]

Supplementary Table 1. Multivariable analysis of good neurological outcomes at 6 months.

| Variables | Adjusted OR (95% CI) | P |
| --- | --- | --- |
| Age, years | 0.959 (0.940–0.978) | <0.001 |
| Male | 2.427 (1.220–4.827) | 0.011 |
| Body mass index, kg/m^2^ | 1.085 (0.999–1.179) | 0.053 |
| Coronary artery disease | 2.073 (0.937–4.583) | 0.072 |
| Diabetes | 0.547 (0.274–1.093) | 0.087 |
| Previous pulmonary disease | 0.684 (0.200–2.337) | 0.545 |
| Previous renal disease | 1.692 (0.568–5.043) | 0.345 |
| Bystander CPR | 0.744 (0.383–1.447) | 0.384 |
| Cardiac etiology | 2.495 (1.290–4.825) | 0.007 |
| SOFA score | 0.886 (0.801–0.980) | 0.019 |
| rCAST | 0.733 (0.683–0.787) | <0.001 |

CPR, cardiopulmonary resuscitation; SOFA, sequential organ failure assessment; rCAST, revised post-Cardiac Arrest Syndrome for Therapeutic hypothermia score; OR, odds ratio; CI, confidence interval.

Supplementary Table 2. Baseline characteristics between the included and excluded patients

| Variables | Inclusion (n = 468) | Exclusion (n = 149) | P |
| --- | --- | --- | --- |
| Demographics |  |  |  |
| Age (years), median (IQR) | 61.3 (49.3–71.8) | 63.2 (51.9–75.2) | 0.122 |
| Male, n (%) | 331 (70.7) | 106 (71.1) | 0.999 |
| Body mass index (kg/m2), median (IQR) | 23.5 (21.3–25.6) | 24.0 (20.9–26.1) | 0.288 |
| Preexisting illness, n (%) |  |  |  |
| Coronary artery disease | 60 (12.8) | 19 (12.8) | 0.999 |
| Arrythmia | 24 (5.1) | 9 (6.0) | 0.824 |
| Congestive heart failure | 21 (4.5) | 3 (2.0) | 0.264 |
| Hypertension | 202 (43.2) | 59 (39.6) | 0.502 |
| Diabetes | 142 (30.3) | 48 (32.2) | 0.742 |
| Stroke | 40 (8.5) | 8 (5.4) | 0.278 |
| Previous pulmonary disease | 38 (8.1) | 11 (7.4) | 0.908 |
| Previous renal disease | 42 (9.0) | 18 (12.1) | 0.339 |
| Liver cirrhosis | 8 (1.7) | 2 (1.3) | 0.999 |
| Malignancy | 22 (4.7) | 15 (10.1) | 0.027 |
| Cardiac arrest characteristics |  |  |  |
| Witnessed collapse, n (%) | 302 (64.5) | 93 (62.4) | 0.711 |
| Bystander CPR, n (%) | 321 (68.6) | 94 (63.1) | 0.252 |
| Shockable rhythm, n (%) | 161 (34.4) | 41 (27.5) | 0.144 |
| Cardiac etiology, n (%) | 257 (54.9) | 90 (60.4) | 0.280 |
| Time from collapse to ROSC (min), median (IQR) | 29.0 (17.0–46.0) | 35.0 (18.5–48.5) | 0.084 |
| Lactate after ROSC (mmol/L), median (IQR) | 9.2 (5.9–12.2) | 10.5 (6.9–13.0), 11 | 0.052 |
| SOFA score | 11 (9–13) | 12 (10–13), 2 | 0.063 |
| Poor neurological outcome, n (%) | 336 (71.8) | 110 (76.9) | 0.271 |

IQR, interquartile range; CPR, cardiopulmonary resuscitation; SOFA, sequential organ failure assessment; rCAST, revised post-Cardiac Arrest Syndrome for Therapeutic hypothermia score; OR, odds ratio; CI, confidence interval.

Supplementary Table 3. Comparison of the baseline characteristics based on neurological outcomes at 6 months in each rCAST severity group.

|  | Low severity | |  | Moderate severity | |  | High severity | |  |
| --- | --- | --- | --- | --- | --- | --- | --- | --- | --- |
| Variables | Good (n = 57) | Poor (n = 14) | P | Good (n = 67) | Poor (n = 148) | P | Good (n = 8) | Poor (n = 174) | P |
| Demographics |  |  |  |  |  |  |  |  |  |
| Age (years), median (IQR) | 56.7 (49.4–63.3) | 64.0 (48.1–77.9) | 0.069 | 53.8 (44.4–66.5) | 66.7 (53.3–76.3) | <0.001 | 49.1 (44.4–60.0) | 62.0 (47.2–72.4) | 0.097 |
| Male, n (%) | 49 (86.0) | 9 (64.3) | 0.135 | 56 (83.6) | 97 (65.5) | 0.011 | 6 (75.0) | 114 (65.5) | 0.864 |
| Body mass index (kg/m2), median (IQR) | 24.0 (22.1–25.8) | 20.8 (18.0–23.9) | 0.014 | 24.2 (22.5–26.8) | 22.7 (20.8–25.3) | 0.002 | 26.1 (25.2–27.3) | 23.5 (20.4–25.2) | 0.010 |
| Preexisting illness, n (%) |  |  |  |  |  |  |  |  |  |
| Coronary artery disease | 12 (21.1) | 2 (14.3) | 0.845 | 12 (17.9) | 21 (14.2) | 0.619 | 2 (25.0) | 11 (6.3) | 0.192 |
| Arrhythmia | 4 (7.0) | 0 (0.0) | 0.709 | 3 (4.5) | 7 (4.7) | 1.000 | 2 (25.0) | 8 (4.6) | 0.092 |
| Congestive heart failure | 4 (7.0) | 1 (7.1) | 0.999 | 1 (1.5) | 11 (7.4) | 0.151 | 0 (0.0) | 4 (2.3) | 0.999 |
| Hypertension | 20 (35.1) | 3 (21.4) | 0.509 | 27 (40.3) | 76 (51.4) | 0.175 | 4 (50.0) | 72 (41.4) | 0.907 |
| Diabetes | 12 (0.0) | 2 (14.3) | 0.845 | 11 (16.4) | 58 (39.2) | 0.002 | 2 (25.0) | 57 (32.8) | 0.942 |
| Stroke | 2 (3.1) | 3 (15.8) | 0.137 | 6 (9.0) | 17 (11.5) | 0.751 | 0 (0.0) | 13 (7.5) | 0.920 |
| Previous pulmonary disease | 1 (1.8) | 3 (21.4) | 0.027 | 4 (6.0) | 14 (9.5) | 0.555 | 0 (0.0) | 16 (9.2) | 0.795 |
| Previous renal disease | 1 (1.8) | 0 (0.0) | 0.999 | 5 (7.5) | 20 (13.5) | 0.293 | 1 (12.5) | 15 (8.6) | 0.999 |
| Liver cirrhosis | 0 (0.0) | 2 (14.3) | 0.046 | 1 (1.5) | 1 (0.7) | 0.999 | 0 (0.0) | 4 (2.3) | 0.999 |
| Malignancy | 3 (5.3) | 1 (7.1) | 0.999 | 3 (4.5) | 9 (6.1) | 0.878 | 0 (0.0) | 6 (3.4) | 0.999 |
| Cardiac arrest characteristics |  |  |  |  |  |  |  |  |  |
| Witnessed collapse, n (%) | 51 (89.5) | 14 (100.0) | 0.464 | 42 (62.7) | 112 (75.7) | 0.073 | 3 (37.5) | 80 (46.0) | 0.914 |
| Bystander CPR, n (%) | 40 (70.2) | 11 (78.6) | 0.769 | 52 (77.6) | 115 (77.7) | 0.999 | 5 (62.5) | 98 (56.3) | 0.999 |
| Shockable rhythm, n (%) | 50 (87.7) | 7 (50.0) | 0.005 | 46 (68.7) | 34 (23.0) | <0.001 | 2 (25.0) | 22 (12.6) | 0.634 |
| Cardiac etiology, n (%) | 50 (87.7) | 8 (57.1) | 0.024 | 51 (76.1) | 81 (54.7) | 0.005 | 5 (62.5) | 62 (35.6) | 0.244 |
| Time from collapse to ROSC (min), median (IQR) | 15.0 (12.0–19.5) | 18.0 (13.3–29.5) | 0.262 | 20.0 (12.0–28.0) | 25.0 (17.0–43.0) | 0.002 | 29.0 (11.8–55.8) | 42.5 (30.0–54.3) | 0.198 |

|  | Low severity | |  | Moderate severity | |  | High severity | |  |
| --- | --- | --- | --- | --- | --- | --- | --- | --- | --- |
| Variables | Good (n = 57) | Poor (n = 14) | P | Good (n = 67) | Poor (n = 148) | P | Good (n = 8) | Poor (n = 174) | P |
| Lactate after ROSC (mmol/L), median (IQR) | 5.2 (3.5–8.1) | 4.2 (2.9–6.1) | 0.230 | 7.4 (5.1–9.9) | 8.2 (5.9–10.5) | 0.249 | 12.8 (7.4–17.5) | 12.1 (9.9–15.0) | 0.685 |
| SOFA score | 9 (7–11) | 11 (8–13) | 0.068 | 10 (8–12) | 11 (9–13) | 0.005 | 11 (7–12) | 12 (11–14) | 0.129 |
| rCAST | 3 (2–4) | 5 (2–6) | 0.065 | 10 (8–11) | 12 (10–13) | <0.001 | 16 (15–18) | 16 (16–18) | 0.414 |
| Duration of MAP >80 mmHg |  |  |  |  |  |  |  |  |  |
| During 0–48 h (hour), median (IQR) | 36 (25–41) | 25 (13–38) | 0.052 | 34 (27–42) | 27 (17–39) | <0.001 | 41 (31–45) | 30 (16–39) | 0.032 |
| During 0–24 h (hour), median (IQR) | 18 (13–21) | 13 (9–20) | 0.113 | 17 (13–21) | 15 (9–20) | 0.048 | 21 (16–24) | 16 (11–20) | 0.055 |
| During 25–48 h (hour), median (IQR) | 18 (13–22) | 12 (3–20) | 0.048 | 18 (13–22) | 12 (5–20) | <0.001 | 22 (12–23) | 12 (4–21) | 0.077 |

CPR, cardiopulmonary resuscitation; ROSC, return of spontaneous circulation; SOFA, sequential organ failure assessment; rCAST, revised post-Cardiac Arrest Syndrome for Therapeutic hypothermia score; MAP, mean arterial pressure.
